# Immunohistochemical staining patterns of alpha-keratins in normal tissues from two reptile species: implications for characterization of squamous cell carcinomas

**DOI:** 10.1186/s12917-018-1545-6

**Published:** 2018-07-06

**Authors:** Jorge Orós, María López-Yánez, Francisco Rodríguez, Pascual Calabuig, Pedro L. Castro

**Affiliations:** 1 Department of Morphology, Veterinary Faculty, University of Las Palmas de Gran Canaria, Trasmontaña s/n, 35413 Arucas, Las Palmas Spain; 2 Tafira Wildlife Rehabilitation Centre, Cabildo de Gran Canaria, Tafira Baja, 35017 Las Palmas de Gran Canaria, Spain

**Keywords:** Alpha-keratin, Bearded dragon, *Caretta caretta*, Immunohistochemistry, Loggerhead Sea turtle, *Pogona vitticeps*, Reptile, Squamous cell carcinoma

## Abstract

**Background:**

Cytokeratins with epitopes in common with those of alpha (acidic and basic) mammalian keratins have been immunohistochemically demonstrated in the epidermis of reptiles. However, there are no reports of immunohistochemical staining patterns of alpha-keratins in other tissues from reptiles. Because the epithelial tumours usually retain the keratin patterns of their normal epithelial origin, it is necessary to know in advance these patterns in the major normal epithelia and organs. We used anti-alpha human keratin AE1 and AE3 monoclonal antibodies to study the staining patterns of alpha-keratins in the major normal epithelia and organs from two reptile species [the bearded dragon (*Pogona vitticeps*) and the loggerhead sea turtle (*Caretta caretta*)]. We also studied the immunolocalization of alpha-keratins in squamous cell carcinomas (SCCs) in a bearded dragon and two loggerhead turtles.

**Results:**

Acidic alpha-keratin (AE1 positive) was detected in many of the epithelial tissues of the bearded dragons; however, the detection of basic alpha-keratin (AE3 positive) was much more limited. Alpha-keratins were detected in a greater number of tissues of loggerhead turtles compared with those observed in bearded dragons. In the bearded dragon SCC, all layers of the nests of neoplastic cells, including the cornified layer of the keratin pearls, were strongly reactive with the AE1 antibody. However, a weak reactivity using the AE3 antibody was detected in the basal and intermediate layers of these nests. In the cutaneous SCCs of both sea turtles, acidic alpha-keratin was detected in the basal and suprabasal layers, and in all of the invasive neoplastic cords, while basic alpha-keratin was mainly detected in the invasive neoplastic cords. The pattern observed in the metastases in both turtles consisted of immunohistological detection of acidic alpha-keratin in all metastatic foci, and limited or lack of detection of basic alpha-keratin.

**Conclusions:**

This study provides, for the first time, information about the immunohistochemical staining patterns of alpha-keratins in normal tissues from bearded dragons and loggerhead sea turtles, and confirms the usefulness of AE1 and AE3 monoclonal antibodies in these reptile species. The use of these antibodies also contributed to a better characterization of SCCs in these species.

## Background

Alpha keratins are typical intermediate filaments of epithelia in mammals. They are formed by pairing of type I (acidic) and type II (basic) molecules and are expressed in highly specific patterns related to the epithelial type and stage of cellular differentiation [1]. Epithelial tumours, including metastases, most widely retain the keratin patterns of their (normal) epithelial origin; thus, the determination of the keratin patterns of tumours is essential for cell and tumour typing [1]. Immunohistochemistry is a valuable tool for the detection of cytokeratins in normal tissues from mammals [2–4] and also for the accurate diagnosis of numerous epithelial tumours in mammals [5–8] and birds [9, 10].

Cytokeratins with epitopes in common with those of acidic and basic mammalian keratins are also present in the epidermis of reptiles [11]. In fact, use of immunocytochemistry for studying the keratinization of soft and hard epidermis using the broadly characterized anti-alpha human keratin AE1, AE2, and AE3 antibodies has been reported in chelonians [12, 13], lizards [14, 15], snakes [16], crocodilians [17], and the tuatara [11]. However, there are no reports of immunohistochemical staining patterns of alpha-keratins in other normal tissues and organs from reptiles.

Squamous cell carcinoma (SCC) is a malignant tumour with low overall prevalence in reptiles [18–21]. SCC has been reported in several species of reptiles, including snakes [20, 22–24], lizards [18, 20, 21, 25], chelonians [20, 26, 27], crocodilians [28], and the tuatara (*Sphenodon punctatus*) [29]. Metastases have been rarely reported [20, 22, 26, 28].

SCC diagnosis in reptiles is based mainly on the histological characteristics [18, 19, 21]. Immunohistochemical characterization of neoplastic cells in reptiles has been reported rarely due to the lack of cross-reactive commercial antibodies [30–33]. In fact, the only attempt to characterize SCCs in reptiles failed [26].

The aims of this study were a) to describe the immunohistochemical staining patterns of alpha-keratins in the major normal epithelia and organs from two reptile species [the bearded dragon (*Pogona vitticeps*) and the loggerhead sea turtle (*Caretta caretta*)], and b) to document the immunolocalization of alpha-keratins in SCCs in these species to contribute to a better characterization of these tumours.

## Methods

### Samples

Normal tissues and organs (Table 1) from four bearded dragons and six loggerhead turtles were used; animals had died due to different diseases and only tissues without lesions were selected. Loggerhead turtles were submitted from the Tafira Wildlife Rehabilitation Centre (TWRC) in 1998, 2005, 2009, 2013, 2016, and 2017. Five turtles had stranded at different points on the coast of Gran Canaria Island and a turtle had been found stranded dead in Fuerteventura Island. The turtles stranded in Gran Canaria died during the stay at the TWRC and necropsies were performed 4–24 h after death. The necropsy of the turtle stranded in Fuerteventura was performed 24 h after finding. Selected bearded dragons were submitted from two different private collections in 2005, 2008, 2011, and 2014; tissue samples were taken 12–24 h after death. The samples from both reptile species were fixed during 24–36 h. Three SCCs were also included: the first one was a SCC diagnosed in 2014 in a two-years-old male bearded dragon after surgical removal of a mass on the lower periorbital region of the left eye; the tumour consisted of nests of neoplastic epithelial cells with stratified squamous differentiation and numerous keratin pearls. The neoplastic cells were noted to have moderate anisokaryosis. The mitotic count was approximately 1 to 2 per high-power field. No vascular invasion was noted in the sections examined. The histological characteristics of the SCCs diagnosed in the two loggerhead turtles were previously reported [26]. Briefly, the first turtle showed a multiple well-differentiated cutaneous SCC with metastases to the ventricular myocardium, lungs, and kidneys, and the second turtle showed a poorly differentiated cutaneous SCC with metastases to heart, lungs, kidneys, spleen, liver, and muscle tissue.

**Table 1:** Immunoreactivity of normal tissues from two reptile species [bearded dragon (*Pogona vitticeps*) and loggerhead sea turtle (*Caretta caretta*)] with commercially available monoclonal anti-alpha human keratin AE1 and AE3 antibodies

	*Pogona vitticeps*		*Caretta caretta*	
	AE1	AE3	AE1	AE3
Epidermis (*n* = 3/4)^a^				
Basal cells	–	+	+++	++
Suprabasal cells	–	+	+++	+++
Pre-cornified layers	+++/++^b^	+	–	++
Cornified layer	+++^c^	–	–	–
Lingual epithelium (*n* = 0/4)	ND	ND	+++^d^	+^d^
Oesophageal epithelium (*n* = 0/6)	ND	ND	+++^d^	+++^e^
Gastric mucosa (*n* = 4/6)				
Surface epithelium	++	–	+++/−^f^	+
Gastric glands	+/−	–	–	–
Small intestine epithelium (*n* = 4/6)	+++	–	+++	–
Large intestine epithelium (*n* = 4/6)	–	–	–	–
Liver (*n* = 4/6)				
Hepatocytes	++	–	++	–
Biliary ductal epithelium	+++	–	+++	–
Gallbladder epithelium (*n* = 0/3)	ND	ND	+	–
Pancreas (*n* = 2/5)				
Acinar epithelium	–	–	–	–
Ductal epithelium	–	–	–	–
Tracheal epithelium (*n* = 4/6)	++	–	+++	–
Lung (bronchial, bronchiolar and faveolar epithelia) (*n* = 4/6)	++	–	+++	–
Kidney (*n* = 4/6)				
Parietal layer of Bowman’s capsule	–	–	+++	+/−
Proximal tubules	–	–	–	–
Distal tubules	++	–	+++	+++
Collecting ducts	+	–	+++	+++
Urinary bladder epithelium (*n* = 0/3)	NA	NA	+/−	–
Salt gland (*n* = 0/6)	NA	NA		
Secretory acini			+++/+^g^	+/−
Ductal epithelium			+++	+++
Thyroid gland epithelium (*n* = 2/5)	–	–	–	–
Spleen (*n* = 4/6)	–	–	–	–
Epicardium (*n* = 4/6)	+	–	+	–
Vascular endothelium (*n* = 4/6)	–	–	–	+/−

Reactivity was judged as intense (+++), moderate (++), weak (+), negative (−), or variable (weak/negative) (+/−), respectively

*ND* not done due to lack of samples

*NA* not applicable (the species does not have this organ)

^a^Number of cases for each individual organ or tissue (*n* = x/y), where ‘x’ stands for the number of tissues from bearded dragons and ‘y’ for that of loggerhead sea turtles

^b^Reactivity within the cytoplasm of suprabasal cells incorporated into the alpha-layer

^c^Reactivity limited to the inner cornified layer (alpha-layer)

^d^Reactivity limited to the basal and suprabasal cells of the epithelium

^e^Reactivity in all layers of the epithelium (except in the cornified layer) but more intense in the suprabasal cells

^f^Reactivity was detected in the majority of the epithelial cells, but some cells were negative

^g^Intense reactivity in the peripheral secretory acini and weak reactivity in the others

### Immunohistochemistry

Immunohistochemistry was performed on formalin-fixed and paraffin wax-embedded tissue samples. Sequential 3 μm sections were dewaxed in xylene and digested with a solution of 0.05% protease type XIV (P-5147; Sigma-Aldrich, Munich, Germany) for five min at 37 °C for antigen retrieval. The sections were then incubated with peroxidase-blocking solution (S2001; Dako, Carpinteria, California, USA) for 10 min and washed three times in phosphate-buffered saline with Tween-20 (PBST). The anti-alpha-keratin type I (acidic) mouse monoclonal (IgG 1 isotype) AE1 antibody (#61804; Progen Biotechnik, Heidelberg, Germany) was applied at 0.002 mg/mL (dilution 1:500), the anti-alpha-keratin type II (basic) mouse monoclonal (IgG 1 isotype) AE3 antibody (#61806; Progen Biotechnik, Heidelberg, Germany) was applied at 0.001 mg/mL (dilution 1:1000), and all samples were incubated overnight at room temperature. Visualisation of bound primary antibody was carried out using the Dako EnVision+ (K4001; Dako, Carpinteria, California, USA) for 60 min, and the diaminobenzidine Substrate-Chromogen System (K3466; Dako, Carpinteria, California, USA), with three changes of PBST between each step. Slides were counterstained with Harris haematoxylin (Fisher Scientific, Loughborough, UK), dehydrated, cleared, and covered with a coverslip. Normal skin samples from dog and cat origin were used as positive controls. Negative controls consisted of a) omitting the primary antibody, and b) replacing the primary antibodies with the anti-glial fibrillary acidic protein mouse monoclonal (IgG 1 isotype) antibody (#G-3893; Sigma, Saint Louis, Missouri) at identical dilutions, on normal skin samples from both reptile species and dog. Each slide was examined microscopically for evidence of reactivity of the primary antisera. A score of (+ + +) was used to indicate intense reactivity, (+ +) for moderate reactivity, (+) for weak reactivity, and (−) for no reactivity.

## Results

**Fig. 1:**
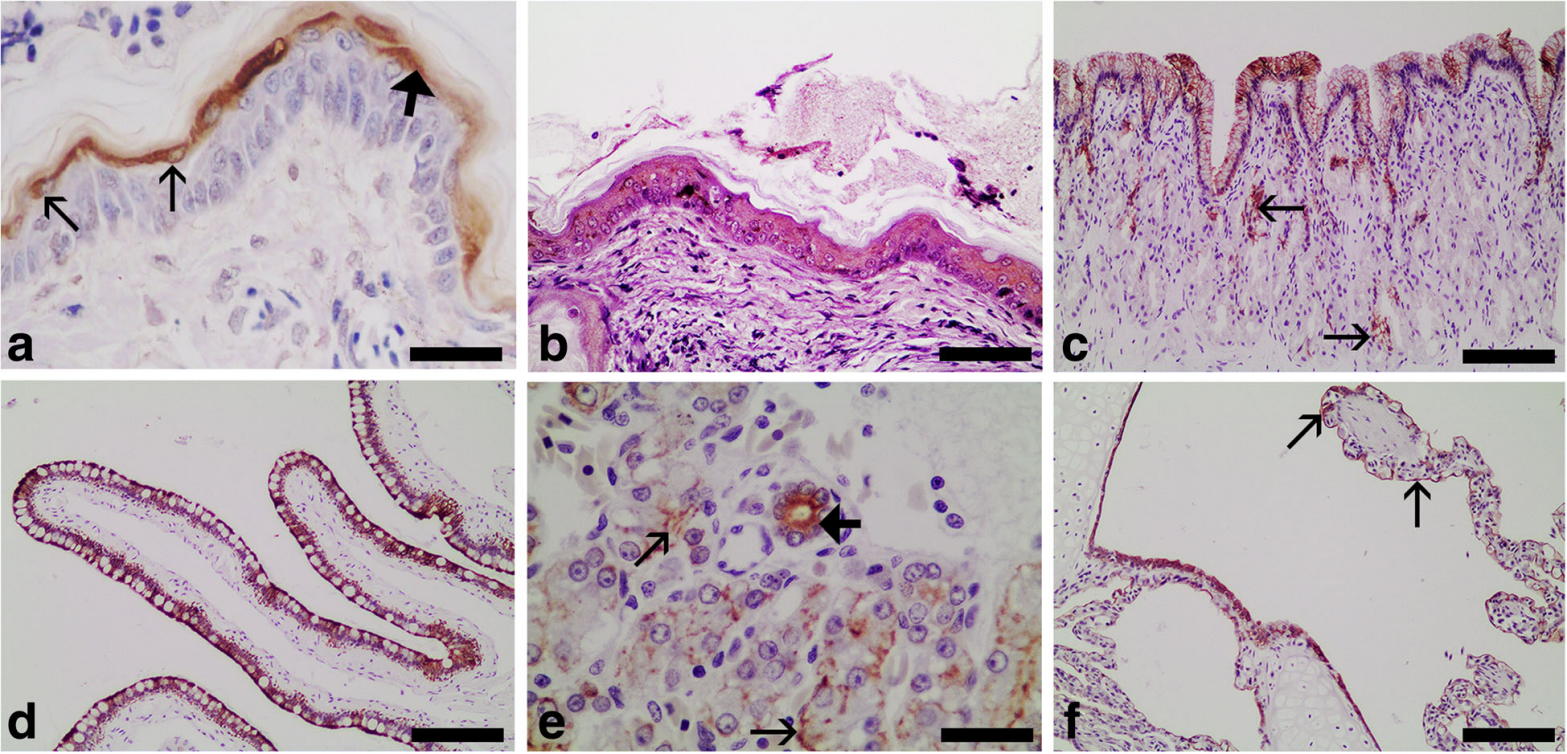
Immunohistochemical detection of acidic alpha-keratin (AE1 positive) (**a**, **c**-**f**) and basic alpha-keratin (AE3 positive) (**b**) in normal tissues from bearded dragons (*Pogona vitticeps*). **a** Epidermis; note the intense immunoreactivity detected in the inner cornified layer (alpha-layer) (bold arrow); note also the intracytoplasmic immunoreactivity in some suprabasal cells incorporated into the alpha-layer (thin arrows). Bar = 40 μm. **b** Epidermis; note the weak immunoreactivity detected in all layers except the cornified layer. Bar = 60 μm. **c** Gastric mucosa showing moderate immunoreactivity in the surface epithelium; only some gastric glands (arrows) showed immunoreactivity. Bar = 120 μm. **d** Small intestine epithelium showing intense immunoreactivity. Bar = 120 μm. **e** Liver; note the moderate immunoreactivity in the hepatocytes (thin arrows) and the intense immunoreactivity in a biliary duct (bold arrow). Bar = 40 μm. **f** Lung; moderate immunoreactivity in the respiratory epithelia, included the faveolar epithelium (arrows). Bar = 120 μm. Dako EnVision+ System, Harris haematoxylin counterstain

**Fig. 2:**
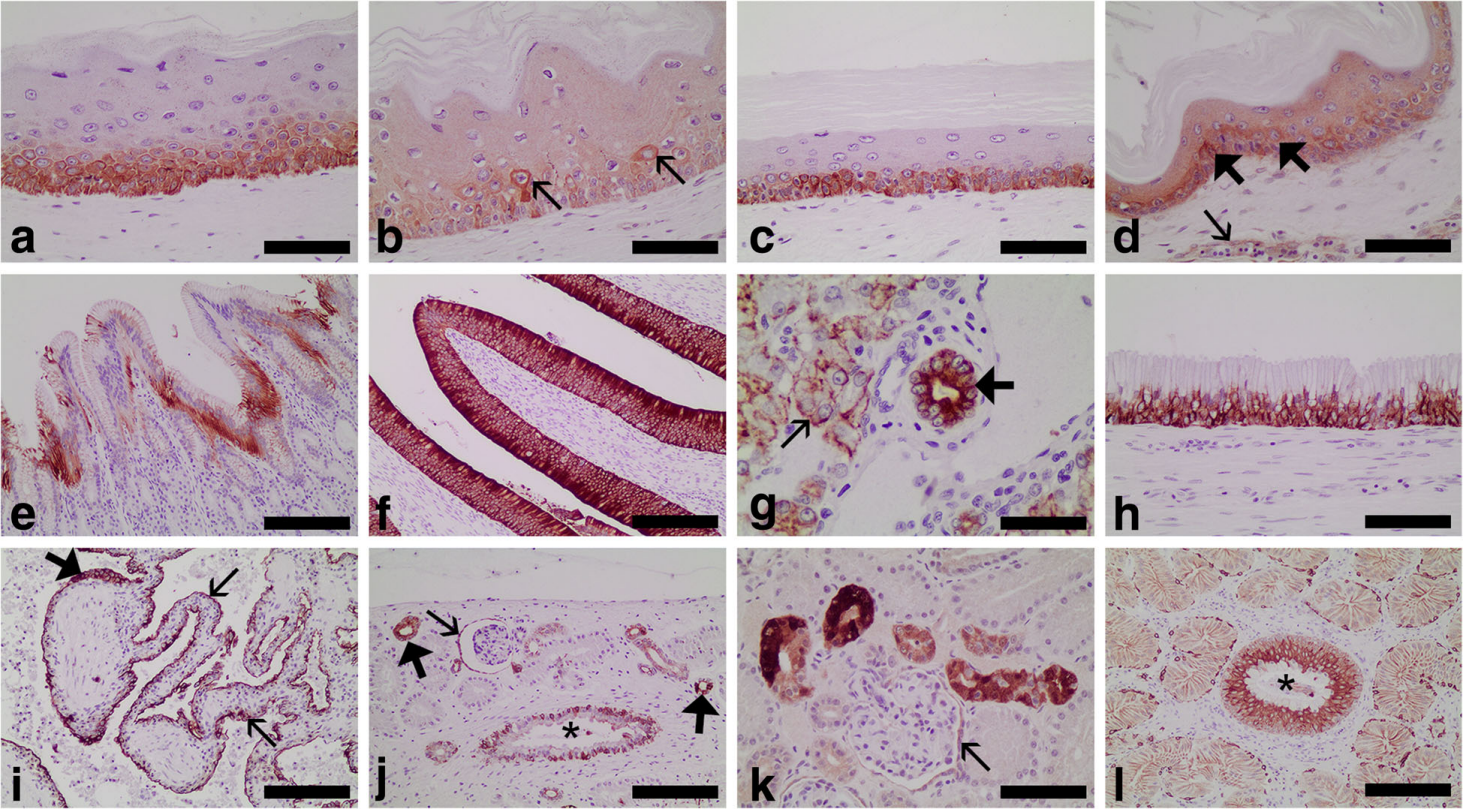
Immunohistochemical detection of acidic alpha-keratin (AE1 positive) (**a**, **c**, **e**-**j**, **l**) and basic alpha-keratin (AE3 positive) (**b**, **d**, **k**) in normal tissues from loggerhead sea turtles (*Caretta caretta*). **a** Epidermis showing intense immunoreactivity in the basal and suprabasal cells. Bar = 70 μm. **b** Epidermis; note that all layers except the cornified layer were immunolabelled, but the most intense immunoreactivity was observed in some suprabasal cells (arrows). Bar = 70 μm. **c** Oesophageal epithelium showing intense immunoreactivity in the basal and suprabasal cells. Bar = 70 μm. **d** Oesophageal epithelium showing immunoreactivity in all layers of the epithelium (except in the cornified layer) but more intense in some suprabasal cells (bold arrows); note also the weak immunoreactivity in the vascular endothelium (thin arrow). Bar = 70 μm. **e** Gastric mucosa showing intense immunoreactivity in many of the lining epithelial cells. Bar = 140 μm. **f** Small intestine epithelium showing intense immunoreactivity. Bar = 140 μm. **g** Liver; note the moderate immunoreactivity in the hepatocytes (thin arrow) and the intense immunoreactivity in a biliary duct (bold arrow). Bar = 35 μm. **h** Tracheal epithelium showing intense immunoreactivity. Bar = 70 μm. **i** Lung; intense immunoreactivity in the faveolar epithelium (thin arrows) and in the epithelium lining the bundles of smooth muscle (bold arrow). Bar = 240 μm. **j** Kidney; immunoreactivity was detected in the parietal layer of Bowman’s capsule (thin arrow), distal tubules (bold arrows), and collecting ducts (*). Bar = 140 μm. **k** Kidney; immunoreactivity was weak in the parietal layer of Bowman’s capsule (thin arrow) and intense in the distal tubules. Bar = 70 μm. **l** Salt gland; intense immunoreactivity in the epithelial cells of a central duct (*) and weak immunoreactivity in adjacent secretory acini. Bar = 140 μm. Dako EnVision+ System, Harris haematoxylin counterstain

The results of the immunohistochemical study for detection of acidic alpha-keratin (AE1 positive) and basic alpha-keratin (AE3 positive) in normal tissues from the reptile species investigated are given in Table 1. Immunoreaction was not observed in the negative controls in any of the studies. Both antibodies presented a similar pattern of intracytoplasmic immunostaining characterized by a predominantly diffuse and homogeneously distributed reaction. Apical or peripheral membranous immunolabelling was occasionally enhanced. Regarding the normal tissues collected from bearded dragons, the most intense reactivity using the monoclonal anti-alpha human keratin AE1 antibody was limited to the inner cornified (alpha-layer) and pre-cornified layers of the epidermis and the epithelial linings of the small intestine and the biliary ducts; a moderate reactivity was observed in the gastric mucosa (surface epithelium), hepatocytes, respiratory epithelia, and the renal distal tubular cells (Fig. 1). Reactivity using the monoclonal anti-alpha human keratin AE3 antibody was only detected in the basal, suprabasal and pre-cornified layers of the epidermis and it was week (Fig. 1b). Acidic alpha-keratin (AE1 positive) and basic alpha-keratin (AE3 positive) were detected in a greater number of tissues of loggerhead turtles compared with those observed in bearded dragons. The most intense reactivity using the AE1 antibody was observed in the basal and suprabasal cells of the epidermis, lingual epithelium and oesophageal epithelium, and in the epithelial linings of the stomach, small intestine, biliary ducts, trachea, lung, kidney (with the exception of the proximal tubules), and salt glands (particularly in the peripheral secretory units and ducts) (Fig. 2). An intense reactivity using the AE3 antibody was detected in the suprabasal cells of the epidermis (Fig. 2b), the basal and suprabasal cells of the oesophageal epithelium (Fig. 2d), the renal distal tubules (Fig. 2k) and collecting ducts, and the ductal epithelium of the salt glands. A weak reactivity using the AE3 antibody was observed in some vascular endothelial cells (Fig. 2d).

**Fig. 3:**
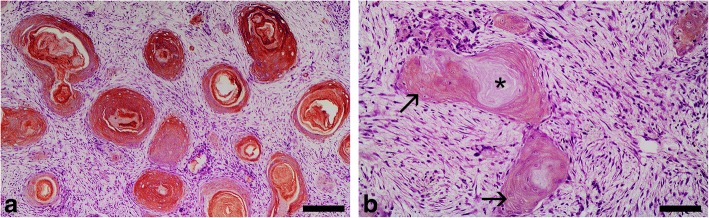
Squamous cell carcinoma, skin, bearded dragon (*Pogona vitticeps*). **a** Immunohistochemistry for acidic alpha-keratin (AE1 positive) showing intense immunoreactivity in all layers of the nests of neoplastic cells, including the cornified layer of the keratin pearls. Bar = 100 μm. **b** Immunohistochemistry for basic alpha-keratin (AE3 positive) showing weak immunoreactivity in the basal and intermediate layers of the nests of neoplastic cells (arrows); no reactivity was observed in the cornified layer of the keratin pearls (*). Bar = 60 μm. Dako EnVision+ System, Harris haematoxylin counterstain

In the bearded dragon SCC, all layers of the nests of neoplastic cells, including the cornified layer of the keratin pearls, were strongly reactive with the AE1 antibody (Table 2, Fig. 3a). In contrast, using the AE3 antibody a weak reactivity was detected in the basal and intermediate layers of the nests of neoplastic cells, and no reactivity was observed in the cornified layer of the keratin pearls (Fig. 3b). Adjacent areas of normal epidermis showed a similar pattern to that reported for the epidermis in the study on normal tissues.

**Table 2:** Immunoreactivity of squamous cell carcinomas (SCCs) in three reptiles [a bearded dragon (*Pogona vitticeps*) and two loggerhead sea turtles (*Caretta caretta*)] with commercially available monoclonal anti-alpha human keratin AE1 and AE3 antibodies

	*Pogona vitticeps*		*C. caretta* #1^a^		*C. caretta* # 2^b^	
	AE1	AE3	AE1	AE3	AE1	AE3
Cutaneous SCC nests						
Basal layer	+++	+	+++	+++	+++	+++
Suprabasal (intermediate) layer	+++	+	+++	+++	+++	+++
Keratin pearls (cornified layer)	+++	–	–	–	–	–
Invasive neoplastic cords (dermis)			+++	+++	+++	+++
Metastases						
Myocardium			+++	++/−^c^	+++	–
Lung			+++	++/−^d^	+++	–
Kidney			+++	++/−^d^	+++	–
Spleen					+++	–
Liver					+++	–
Muscle tissue					+++	–

Reactivity was judged as intense (+++), moderate (++), weak (+), negative (−), or variable (weak/negative) (+/−), respectively

^a^Well-differentiated metastatic cutaneous SCC

^b^Poorly differentiated metastatic cutaneous SCC

^c^Only the biggest metastatic foci showed immunoreactivity (limited to suprabasal and pre-cornified layers)

^d^Reactivity was detected in a few metastases showing stratified squamous differentiation

**Fig. 4:**
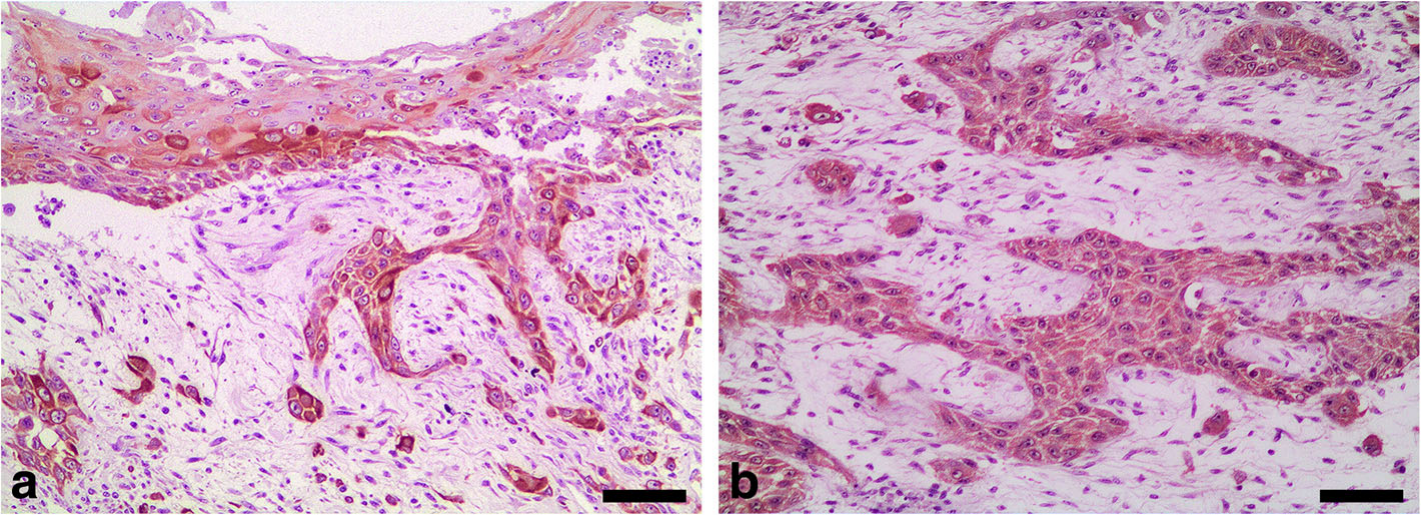
Squamous cell carcinoma, skin, loggerhead sea turtle (*Caretta caretta*) No. 1. **a** Immunohistochemistry for acidic alpha-keratin (AE1 positive) showing intense immunoreactivity in the basal and suprabasal cells, and in all of the invasive neoplastic cords. Bar = 80 μm. **b** Immunohistochemistry for basic alpha-keratin (AE3 positive) showing intense immunoreactivity in the invasive neoplastic cords detected in the dermis. Bar = 65 μm. Dako EnVision+ System, Harris haematoxylin counterstain

In the cutaneous SCCs of both sea turtles, acidic alpha-keratin (AE1 positive) was detected in the basal and suprabasal layers, and in all of the invasive neoplastic cords (Table 2, Fig. 4a); the most intense reactivity using the AE3 antibody was observed in the invasive neoplastic cords detected in the dermis (Fig. 4b). Adjacent areas of normal epidermis showed a similar pattern to that reported for the epidermis in the study on normal tissues.

**Fig. 5:**
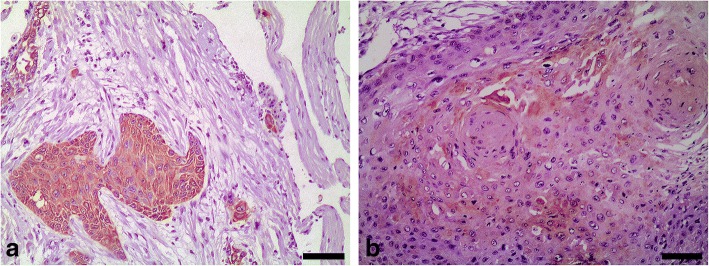
Metastases of squamous cell carcinoma, heart, loggerhead turtle (*Caretta caretta*) No. 2. **a** Immunohistochemistry for acidic alpha-keratin (AE1 positive) showing intense immunoreactivity in all cells of the metastatic foci. Bar = 80 μm. **b** Immunohistochemistry for basic alpha-keratin (AE3 positive) showing intense immunoreactivity only in the suprabasal and pre-cornified layers of a big metastatic focus. Bar = 110 μm. Dako EnVision+ System, Harris haematoxylin counterstain

**Fig. 6:**
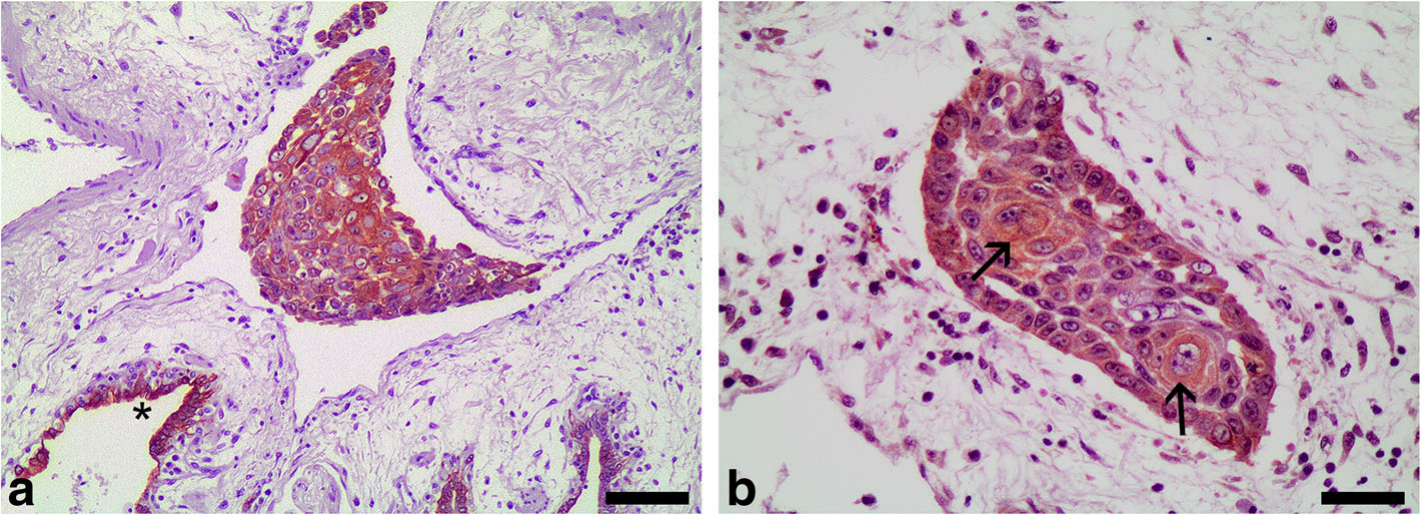
Metastases of squamous cell carcinoma, lung, loggerhead turtle (*Caretta caretta*) No. 1. **a** Immunohistochemistry for acidic alpha-keratin (AE1 positive) showing strong immunoreactivity in all cells of the metastatic foci; note also the labelling of the normal epithelium of the bronchioles (*). Bar = 80 μm. **b** Immunohistochemistry for basic alpha-keratin (AE3 positive) in a small metastatic focus showing stratified squamous differentiation (arrows). Bar = 110 μm. Dako EnVision+ System, Harris haematoxylin counterstain

Acidic alpha-keratin (AE1 positive) was detected in all of the metastases in the myocardium from both turtles (Fig. 5a), whereas basic alpha-keratin (AE3 positive) was only detected in a few metastases in the myocardium of the first turtle. These few metastases were the biggest metastatic foci, and immunoreactivity was limited to suprabasal and pre-cornified layers (Fig. 5b). Similarly, acidic alpha-keratin (AE1 positive) was detected in all the lung metastases from both turtles (Fig. 6a). Basic alpha-keratin (AE3 positive) was detected in a few metastases in the first turtle’s lung, mainly in some foci showing stratified squamous differentiation (Fig. 6b). A similar pattern was detected in the kidney metastases.

All of the small metastatic foci in the spleen from the second turtle showed intense immunoreactivity against acidic alpha-keratin; however, no basic alpha-keratin was detected immunohistologically. A pattern similar to that observed in the spleen was detected in the second turtle’s liver and muscle tissue metastases.

## Discussion

This study provides a catalogue of tissues and cells from bearded dragons and loggerhead sea turtles labelled by anti-alpha human keratin AE1 and AE3 antibodies.

In bearded dragons, acidic alpha-keratin (AE1 positive) was detected (with different intensities) in many of the normal epithelial tissues studied; however, detection of basic alpha-keratin (AE3 positive) was much more limited. Reactivity using the AE1 antibody in the normal tissues from loggerhead sea turtles was generally more intense than that observed in tissues from bearded dragons. The labelling using the AE3 antibody was more limited, although basic alpha-keratin (AE3 positive) was detected in a greater number of tissues compared with those observed in bearded dragons. The differences observed between these two species can be attributed to the fact that they belong to different taxonomic orders (Squamata for the bearded dragon, and Testudines for the loggerhead sea turtle) [34]. In fact, different staining patterns using the anti-alpha human keratin AE1and AE3 antibodies in normal canine tissues compared to those observed in human tissues have also been reported [2]. According to the manufacturer’s instructions, the anti-alpha human keratin (type I) AE1 antibody detects specifically the keratins K19, K16, K14 and most other acidic (type I) keratins, whereas the anti-alpha human keratin (type II) AE3 antibody represents an excellent marker for basic (type II) keratins K1-K8. The cell type and tissue distribution for all human keratins has been studied in depth [1]. However, despite recent advances in the study of keratins in domestic mammals [35], knowledge about specific cell type and tissue distribution for all keratins in other animal species, particularly reptiles, is scarce.

The epidermis is undoubtedly the epithelial tissue most deeply studied in reptiles in terms of the expression of their different keratins [11–17]. Two main types of keratins, alpha and beta, have been described in the reptilian epidermis. Alpha-keratin bundles are formed by intermediate cytokeratin filaments that are derived from the association of one acidic to one basic cytokeratin. The alpha-keratin pattern is formed by 8–10 nm diameter electron-pale filaments among an electron-denser matrix material, resulting in pliable and stretchable mature alpha-keratinocytes. Beta-keratin is different as a result of different amino acidic sequences, which determine a beta-pleated conformation. The beta-keratin pattern is formed by 3–4 nm diameter electron-pale fibrils among a sparse matrix material that results in hard and inelastic mature corneocytes [15]. The distribution of both keratins in the reptilian epidermis varies according to the taxonomic order [36].

There are important differences in the histological structure of the epidermis between mammals and the different reptilian orders. In mammals, the epidermal cells are continuously produced in the basal layer, move suprabasally, keratinize, and are desquamated. In contrast, in lizards and snakes the epidermal cells are not continuously produced (shedding cycle), and an outer epidermal generation composed of six keratinized layers (oberhautchen, beta-, mesos, alpha-, lacunar, and clear) is formed. After shedding, only four keratinized layers (a new oberhautchen, beta-, mesos, and an incomplete alpha-layer) are recognised [14]. Therefore, four histological layers can be identified in the epidermis of lizards in this stage: an external beta-layer, an underlying alpha-layer, a few layers of living suprabasal cells and a basal layer [14]. In addition, it is also possible to detect how some of the suprabasal cells are added to the alpha-layer [14]. The microscopic structure of the soft epidermis of turtles is relatively simple and consists of a stratified, keratinized epithelium lacking a granular layer [12]. Crocodilian epidermis, similar to that of shell scutes in chelonians, consists of a stratified and hard epidermis [17].

The distribution pattern of acidic alpha-keratin (AE1 positive) in the normal skin of the bearded dragons differed from what was reported for the normal resting stage epidermis of the Italian wall lizard (*Podarcis sicula*). The absence of reactivity in the basal and suprabasal cells in the bearded dragons could induce to doubt the specificity of the reaction observed in the cornified layer; however, some suprabasal cells incorporated into the alpha-layer were also labelled, and these cells are still transcriptionally active [14]. The question why the basal and inner suprabasal cells were not labelled should be object of further studies. Of note, this staining pattern was also observed in the normal skin from the bearded dragon with the SCC (in which, conversely, all layers of neoplastic cells showed intense immunoreactivity). In the Italian wall lizard, the AE1 antibody labelled the basal and suprabasal living cells, and the immunoreactivity decreased, or disappeared, in the external keratinized layers, mainly in the beta-layer [14]. In addition, some areas (the hinge regions between the scales) remained completely unlabelled with the AE1 antibody [14]. Alpha-keratin was also detected in the keratinized layer of the outer and inner scale surfaces of the normal epidermis of the Italian wall lizard, but it was labelled with the AE2 antibody [14]. The electrophoretic study suggested that in the Italian wall lizard, keratins with low molecular weight are produced in the lowermost layers, and their molecular weight (MW) increases in the upper layers. In the Italian wall lizard, the AE1 antibody recognised acidic cytokeratins with low (44–45 kDa) or intermediate MW (57–58 kDa); however, in mammals the AE1 antibody recognises acidic cytokeratins with MW of 50 kDa and 57–58 kDa [14]. There are no similar studies describing the distribution and molecular characteristics of cytokeratins in the epidermis of bearded dragons. Our results show that the AE1 antibody did not label the basal and inner suprabasal cells of the normal epidermis. However, the distribution pattern of basic alpha-keratin (AE3 positive) in the normal skin of the bearded dragons was similar to that described for the Italian wall lizard [14].

The distribution pattern of alpha-keratins in the normal skin of the loggerhead sea turtles was very similar to that reported in the shelled and non-shelled epidermis of the painted turtle (*Chrysemys picta*). In that freshwater turtle species, the AE1 antibody labelled mostly the germinal layer, and the AE3 immunofluorescence was present in the suprabasal keratinizing, pre-cornified, and faintly in the cornified layer [12].

Since keratins exhibit characteristic expression patterns in tumours, they have great importance in immunohistochemical tumour diagnosis of carcinomas, in particular of unclear metastases and in precise classification and subtyping [1]. Because the epithelial tumours usually retain the keratin patterns of their normal epithelium of origin, it is necessary to know previously these patterns in the major normal epithelia and organs. Our immunohistochemical study provides, for the first time, information about the staining patterns of alpha-keratins in normal tissues from two reptile species: the bearded dragon, a very popular exotic pet species, and the loggerhead sea turtle, included in the Red List of the World Conservation Union as ‘*Vulnerable*’ [37].

Conversely to the situation in mammals [5–8], there are no reports of immunohistological characterization of reptile SCCs. In the bearded dragon SCC, the distribution pattern of acidic alpha-keratin (AE1 positive) was different to that observed in the normal epidermis. All layers of the nests of neoplastic cells, particularly in those containing keratin pearls, showed intense reactivity with the AE1 antibody, suggesting that neoplastic cells overexpressed acidic alpha-keratins. In humans, overexpression of acidic alpha-keratin 17 has been reported in oral squamous cell carcinomas compared to normal mucosa [38].

In both sea turtles the distribution pattern of alpha-keratins in the cutaneous tumours was similar to that observed in the normal epidermis of loggerheads and painted turtle [12]. While the AE1-positive keratins in mammalian epidermis that have been described are limited to the basal layer, those of painted turtles [12] and the loggerhead sea turtles reported here were less precisely localized but still limited to the most basal layers. The distribution pattern of AE3-positive keratins was also similar to that described in the mammalian epidermis [12].

Regarding the metastases diagnosed in both turtles, the pattern observed repeatedly consisting of immunohistological detection of acidic alpha-keratin (AE1 positive) in all metastatic foci, and limited or lacking detection of basic alpha-keratin (AE3) in these metastatic foci, might be related to poorly differentiated cells, in which some cytoskeleton proteins are not fully developed or have not been preserved during neoplastic transformation; in fact, a poorly differentiated SCC had been diagnosed in the turtle in which inmmunohistological detection of basic alpha-keratin failed.

## Conclusions

This study provides, for the first time, information about the immunohistochemical staining patterns of alpha-keratins in normal tissues from bearded dragons and loggerhead sea turtles, and confirms the usefulness of AE1 and AE3 monoclonal antibodies for detecting alpha-keratins in these reptiles. The use of these antibodies also contributed to a better characterization of SCCs in these species.

## Abbreviations

AE1: Anti-alpha-keratin type I (acidic) monoclonal antibody
AE3: Anti-alpha-keratin type II (basic) monoclonal antibody
MW: Molecular weight
PBST: Phosphate-buffered saline Tween-20 solution
SCC: Squamous cell carcinoma

## References

[1] Moll R, Divo M, Langbein L (2008). The human keratins: biology and pathology. Histochem Cell Biol.

[2] Cardona A, Madewell BR, Naydan DK, Lund JK (1989). A comparison of six monoclonal antibodies for detection of cytokeratins in normal and neoplastic canine tissues. J Vet Diagn Investig.

[3] Chu PG, Weiss LM (2002). Keratin expression in human tissues and neoplasms. Histopathology.

[4] Perrin C, Langbein L, Schweizer J (2004). Expression of hair keratins in the adult nail unit: an immunohistochemical analysis of the onychogenesis in the proximal nail fold, matrix and nail bed. Br J Dermatol.

[5] Yoshikawa H, Ehrhart EJ, Charles JB, Thamm DH, LaRue SM (2012). Immunohistochemical characterization of feline oral squamous cell carcinoma. Am J Vet Res.

[6] Rodríguez F, Castro P, Ramírez GA (2016). Collision tumour of squamous cell carcinoma and malignant melanoma in the oral cavity of a dog. J Comp Pathol.

[7] Ramos-Vara JA, Edmonson EF, Miller MA, Dusold DM (2017). Immunohistochemical profile of 20 feline renal cell carcinomas. J Comp Pathol.

[8] Sawa M, Inoue M, Yabuki A, Kohyama M, Miyoshi N, Setoguchi A (2017). Rapid immunocytochemistry for the detection of cytokeratin and vimentin: assessment of its diagnostic value in neoplastic diseases of dogs. Vet Clin Pathol.

[9] Watson VE, Murdock JH, Cazzini P, Schnellbacher R, Divers SJ, Sakamoto K (2013). Retrobulbar adenocarcinoma in an Amazon parrot (*Amazona autumnalis*). J Vet Diagn Investig.

[10] McCleery B, Jones MP, Manasse J, Johns S, Gompf RE, Newman S (2015). Pericardial mesothelioma in a yellow-naped Amazon parrot (*Amazona auropalliata*). J Avian Med Surg.

[11] Alibardi L (2004). Formation of the corneous layer in the epidermis of the tuatara (*Sphenodon punctatus*, Sphenodontida, Lepidosauria, Reptilia). Zoology.

[12] Alibardi L (2002). Immunocytochemical observations on the cornification of soft and hard epidermis in the turtle *Chrysemys picta*. Zoology.

[13] Alibardi L, Toni M (2006). Immunolocalization and characterization of beta-keratins in growing epidermis of chelonians. Tissue Cell.

[14] Alibardi L, Maurizii M, Taddei C (2001). Immunocytochemical and electrophoretic distribution of cytokeratins in the resting stage epidermis of the lizard *Podarcis sicula*. J Exp Zool.

[15] Alibardi L, Spisni E, Frassanito AG, Toni M (2004). Characterization of beta-keratins and associated proteins in adult and regenerating epidermis of lizards. Tissue Cell.

[16] Toni M, Alibardi L (2007). Alpha- and beta-keratins of the snake epidermis. Zoology.

[17] Alibardi L, Toni M (2007). Characterization of keratins and associated proteins involved in the cornification of crocodilian epidermis. Tissue Cell.

[18] Hernandez-Divers SM, Garner MM (2003). Neoplasia of reptiles with an emphasis on lizards. Vet Clin N Am-Exot Anim Pract.

[19] Garner MM, Hernandez-Divers SM, Raymond JT (2004). Reptile neoplasia: a retrospective study of case submissions to a specialty diagnostic service. Vet Clin N Am-Exot Anim Pract.

[20] Sykes JM, Trupkiewicz JG (2006). Reptile neoplasia at the Philadelphia zoological garden, 1901-2002. J Zoo Wildl Med.

[21] Hannon DE, Garner MM, Reavill DR (2011). Squamous cell carcinomas in inland bearded dragons (*Pogona vitticeps*). J Herp Med Surg.

[22] Anderson ET, Kennedy-Stoskopf S, Sandy JR, Dorn B, Boyette T, Harms CA (2010). Squamous cell carcinoma with vascular invasion in a diamondback rattlesnake (*Crotalus adamanteus*). J Zoo Wildl Med.

[23] Steeil JC, Schumacher J, Hecht S, Baine K, Ramsay EC, Ferguson S (2013). Diagnosis and treatment of a pharyngeal squamous cell carcinoma in a Madagascar ground boa (*Boa madagascariensis*). J Zoo Wildl Med.

[24] Eleni C, Corteggio A, Altamura G, Meoli R, Cocumelli C, Rossi G (2017). Detection of papillomavirus DNA in cutaneous squamous cell carcinoma and multiple papillomas in captive reptiles. J Comp Pathol.

[25] Abou-Madi N, Kern TJ (2002). Squamous cell carcinoma associated with a periorbital mass in a veiled chameleon (*Chamaeleo calyptratus*). Vet Ophthalmol.

[26] Orós J, Tucker S, Fernández L, Jacobson ER (2004). Metastatic squamous cell carcinoma in two loggerhead sea turtles *Caretta caretta*. Dis Aquat Org.

[27] Lanza A, Baldi A, Spugnini EP (2015). Surgery and electrochemotherapy for the treatment of cutaneous squamous cell carcinoma in a yellow-bellied slider (*Trachemys scripta scripta*). J Am Vet Med Assoc.

[28] Hill AG, Dennis MM, Pyne M (2016). Squamous cell carcinoma with hepatic metastasis in a saltwater crocodile (*Crocodylus porosus*). Aust Vet J.

[29] Roe WD, Alley MR, Cooper SM, Hazley L (2002). Squamous cell carcinoma in a tuatara (*Sphenodon punctatus*). N Z Vet J.

[30] Petterino C, Bedin M, Podestà G, Ratto A (2006). Undifferentiated tumor in the ovary of a corn snake (*Elaphe guttata guttata*). Vet Clin Pathol.

[31] Ritter JM, Garner MM, Chilton JA, Jacobson ER, Kiupel M (2009). Gastric neuroendocrine carcinomas in bearded dragons (*Pogona vitticeps*). Vet Pathol.

[32] Gál J, Mándoki M (2012). Adenoma of the cloacal scent gland in a California kingsnake (*Lampropeltis getulus californiae*). Acta Vet Hung.

[33] Heckers KO, Aupperle H, Schmidt V, Pees M (2012). Melanophoromas and iridophoromas in reptiles. J Comp Pathol.

[34] Doneley B, Doneley B, Monks D, Johnson R, Carmel B (2018). Taxonomy and introduction to common species. Reptile medicine and surgery in clinical practice.

[35] Balmer P, Bauer A, Pujar S, McGarvey KM, Welle M, Galichet A (2017). A curated catalog of canine and equine keratin genes. PLoS One.

[36] Alibardi L, Toni M (2006). Cytochemical, biochemical and molecular aspects of the process of keratinization in the epidermis of reptilian scales. Prog Histochem Cytochem.

[37] The IUCN red list of threatened species. Version 2017-3. http://www.iucnredlist.org (2017). Accessed 15 Feb 2018.

[38] Kitamura R, Toyoshima T, Tanaka H, Kawano S, Kiyosue T, Matsubara R (2012). Association of cytokeratin 17 expression with differentiation in oral squamous cell carcinoma. J Cancer Res Clin Oncol.

